# The preoperative and the postoperative neutrophil-to-lymphocyte ratios both predict prognosis in gastric cancer patients

**DOI:** 10.1186/s12957-020-02059-4

**Published:** 2020-11-10

**Authors:** Eun Young Kim, Kyo Young Song

**Affiliations:** 1 Department of Surgery, UIjeongbu St. Mary Hospital, College of Medicine, The Catholic University of Korea, Seoul, Korea; 2 Department of Surgery, Seoul St. Mary’s Hospital, College of Medicine, The Catholic University of Korea, Seoul, Korea

**Keywords:** Systemic inflammatory response, Postoperative, Neutrophil-to-lymphocyte ratio, Prognostic factor, Gastric cancer

## Abstract

**Background:**

Both the preoperative and postoperative neutrophil-to-lymphocyte ratios (NLRs) have been proposed to predict the long-term prognosis in some cancers, including gastric cancer. The present study investigated the prognostic impact of postoperative NLR, and its preoperative to postoperative changes, in patients with gastric cancer.

**Methods:**

From 2009 to 2012, 1227 consecutive patients who underwent curative surgery for gastric cancer were enrolled in this study. The optimal cut-off value for the postoperative 6-month NLR was 1.7, as determined by receiver operating characteristic curve analysis. Patients were categorized into low- and high-NLR groups based on their postoperative NLR. Four additional groups (low to low, low to high, high to low, and high to high groups) were defined based on the preoperative to postoperative change in the NLR.

**Results:**

The 5-year overall survival (OS) rates of the low- and high-NLR group were 90.7% and 83.0%, respectively (*P* < 0.001). The differences in OS were significant in stage I and stage III gastric cancer patients (*P*< 0.001 and 0.012, respectively). Postoperative NLR was an independent prognostic factor for OS (hazard ratio [HR] = 1.556; *P* = 0.010). The high to high NLR change was a significant predictor of OS (HR = 1.817; *P* = 0.003).

**Conclusions:**

High preoperative and postoperative NLRs, and especially the persistent elevation of preoperative to postoperative NLR, were significant poor prognostic factors for OS in patients with gastric cancer.

**Supplementary Information:**

**Supplementary information** accompanies this paper at 10.1186/s12957-020-02059-4.

## Introduction

Gastric cancer is the third leading cause of cancer-related death in men, and the fifth leading cause in women [1]. Gastrectomy with lymph node dissection remains the mainstay treatment for gastric cancer [2] but the prognosis differs among patients. This has led to increased interest in individualized therapy based on the specific characteristics of the tumor. The only reliable prognostic indicator at present is the tumor, node, metastasis (TNM) stage; however, as patients with the same tumor stage have a heterogeneous prognosis, additional reliable prognostic factors are needed. Accurate prognostic indicators would improve the early management of gastric cancer patients, especially those with a poor prognosis.

Experimental studies have suggested that systemic inflammatory responses play a crucial role in promoting cancer, via pro-inflammatory molecules produced by innate immune cells. There is increasing evidence that the local immune response and systemic inflammation support tumor progression in patients with established cancer, adversely affecting their survival [3]. Recent investigations have shown a significant relationship between postoperative inflammatory markers, including C-reactive protein (CRP), leukocytosis, thrombocytosis, and elevated neutrophil-to-lymphocyte ratio (NLR) or platelet-to-lymphocyte ratio (PLR), and poor survival in patients with various cancers [4–11]. However, a few studies have examined the association between markers of postoperative inflammation, especially the NLR, and the prognosis of patients with gastric cancer [5, 9–11]. Instead, most studies have focused on preoperative measures [12–18]. Therefore, in this study, we examined the clinical utility of postoperative NLR, and its preoperative to postoperative changes, as prognostic indicators of gastric cancer.

## Patients and methods

Prospectively collected data on 1943 patients with gastric adenocarcinoma who underwent gastrectomy at Seoul St. Mary’s Hospital between 2009 and 2012 were reviewed. Of these patients, 1612 patients underwent curative surgery (R0) for gastric cancer. After exclusion, 1227 patients were enrolled in this study. The exclusion criteria were as follows: remnant gastric cancer, neoadjuvant chemotherapy, synchronous and metachronous malignancies, emergency surgery, liver cirrhosis, evidence of a severe inflammatory condition, coexisting hematological malignancies or disorders, autoimmune disorders and recent steroid therapy, incomplete/inaccurate medical records, and absence of postoperative 6-month routine blood examination. Institutional Review Board approval was obtained for this study (KC17RESI0108).

### Blood sample analysis

Blood samples were obtained 6 months postoperatively for determination of the white blood cell, neutrophil, and lymphocyte counts.

### Determination of the optimum NLR cut-offs

**Fig. 1:**
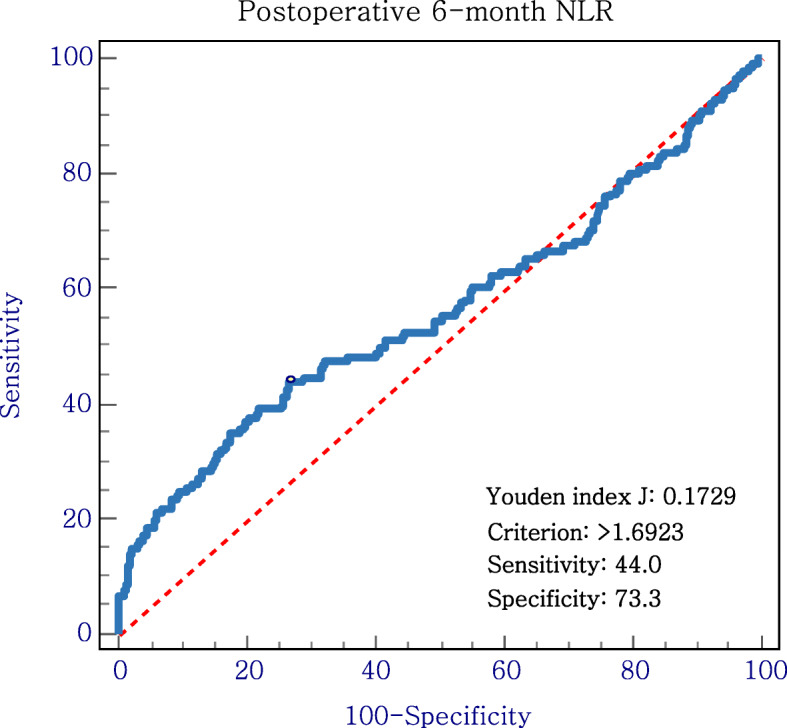
Receiver operating characteristic curves of the neutrophil to lymphocyte ratio (NLR) in gastric cancer patients at 6 months postoperatively (cut-off value, 1.7; sensitivity, 44.0%; specificity, 73.3%)

The NLR was defined as the neutrophil count divided by the lymphocyte count. The optimal cut-off value for the postoperative 6-month NLR was 1.7, as determined by receiver operating characteristic (ROC) curve analysis based on 5-year overall survival (OS) as an endpoint. The area under the ROC curve for the postoperative 6-month NLR was 0.563. Therefore, the sensitivity and specificity of the cut-off value of the NLR for OS were 44.0% and 73.3%, respectively (Fig. 1). The cut-off value of the preoperative NLR was 2.0. This value was used based on our previous study about the prognostic role of the preoperative NLR in gastric cancer [12]. Patients with an NLR above the cut-off value were assigned to the high-NLR (HNLR) group, and the others were assigned to the low-NLR (LNLR) group. In addition, the patients were categorized into four groups based on the preoperative to postoperative change in the NLR: low to low (LL), low to high (LH), high to low (HL), and high to high (HH) groups.

### Statistical analysis

ROC curve analysis was performed to evaluate the sensitivity and specificity of the NLR with respect to 5-year OS, and the Youden index was estimated to determine the optimal NLR cut-off values. Categorical variables were compared using the chi-square test. Kaplan–Meier curves were used to estimate OS and disease-free survival (DFS). The groups were compared in terms of survival using the log-rank test. A Cox regression model was used to identify variables influencing OS and DFS. Then multivariate analyses were performed including all variables showing significant independent relationships with OS and DFS. In all analyses, *P* < 0.05 was taken to indicate statistical significance. All statistical analyses were performed using the SPSS software (version 18.0; SPSS Inc., Chicago, IL, USA).

## Results

### Clinicopathological characteristics

The 1227 patients enrolled in the study had a mean age of 58.4 years old (SD, 11.8) and 811 (66.1%) were male. The open approach method was used in 62.2% cases. Overall, 919 patients (74.9%) underwent partial gastrectomy, and most of the patients (*n* = 804; 65.5%) presented with stage I disease. There were 649 patients (52.9%) with undifferentiated-type cancer.

### Relationship between clinicopathological characteristics and the inflammation-based score

Based on the postoperative 6-month NLR cut-off value, there were 875 LNLR and 352 HNLR patients. Older age, male gender, differentiated cancer, and not receiving the adjuvant chemotherapy were significantly associated with a HNLR (Table 1) (*P <* 0.001, *P* = 0.021, 0.002, and 0.024, respectively).

**Table 1:** Associations of patient characteristics with the postoperative neutrophil-to-lymphocyte ratio (NLR)

Factors	NLR		*P* value
	LNLR (*n* = 875) (%)	HNLR (*n* = 352) (%)	
Age in years, (mean ± SD)	57.5 ± 11.7	60.8 ± 11.7	< 0.001
Gender			0.021
Male	561 (64.1)	250 (71.0)	
Female	314 (35.9)	102 (29.0)	
Approach method			0.636
Open	546 (62.4)	217 (61.7)	
Laparoscopy	310 (35.4)	120 (36.9)	
Robot	19 (2.2)	5 (1.4)	
Extent of resection			0.173
Partial gastrectomy	646 (73.8)	273 (77.6)	
Total gastrectomy	229 (26.2)	79 (22.4)	
Histologic type			0.002
Differentiated	388 (44.3)	190 (54.0)	
Undifferentiated	487 (55.7)	162 (46.0)	
Depth of invasion^a^			0.428
T1	544 (62.2)	219 (62.2)	
T2	87 (9.9)	45 (12.8)	
T3	119 (13.6)	41 (11.7)	
T4	125 (14.3)	47 (13.3)	
Node status^a^			0.141
N0	588 (67.2)	241 (68.5)	
N1	119 (13.6)	35 (9.9)	
N2	89 (10.2)	33 (9.4)	
N3	79 (9.0)	43 (12.2)	
Stage^a^			0.282
I	567 (64.8)	237 (67.3)	
II	157 (17.9)	50 (14.2)	
III	151 (17.3)	65 (18.5)	
Adjuvant chemotherapy			0.024
No	602 (68.8)	265 (75.3)	
Yes	273 (31.2)	87 (24.7)	

*SD* standard deviation

^a^According to the AJCC TNM classification, 7th edition

### Prognostic factors for OS and DFS

**Fig. 2:**
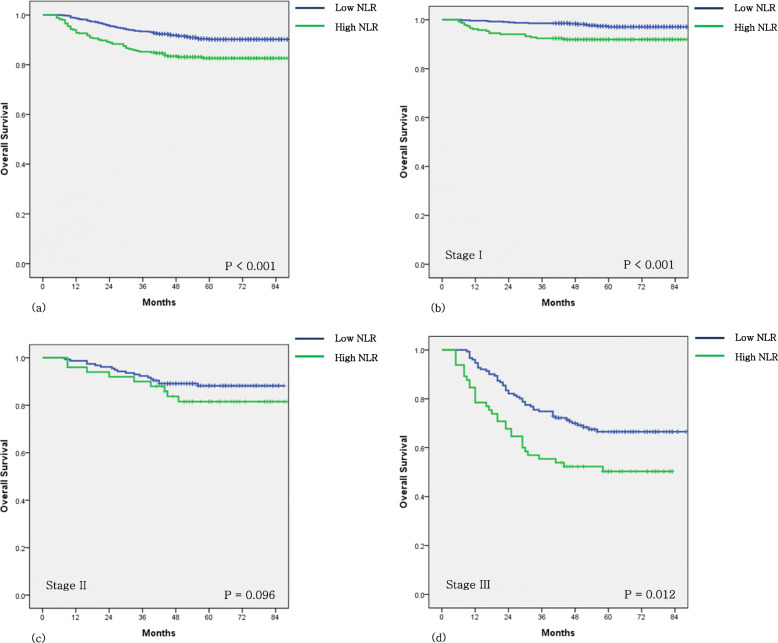
Survival analysis. **a** Overall survival (OS) according to the 6-month postoperative NLR (*P* < 0.001). **b** Patients with stage I gastric cancer: OS according to the NLR at 6 months postoperatively (*P* < 0.001). **c** Patients with stage II gastric cancer: OS according to the NLR at 6 months postoperatively (*P* = 0.096). **d** Patients with stage III gastric cancer: OS according to the NLR at 6 months postoperatively (*P* = 0.012)

The OS of the HNLR group was significantly lower than that of the LNLR group (Fig. 2a) (*P* < 0.001). In univariate analyses, age, approach method, extent of resection, depth of invasion, node status, stage, adjuvant chemotherapy, preoperative NLR, postoperative NLR, and NLR change were significant prognostic factors for OS. In multivariate analyses, preoperative HNLR, postoperative HNLR, and change in HH NLR were significant independent risk factors for poorer OS after adjusting the inflammatory markers for age, approach method, extent of resection, depth of invasion, node status, stage, and adjuvant chemotherapy (Table 2) (*P* = 0.025, 0.010, and 0.003, respectively).

**Table 2:** Univariate and multivariate analyses of factors predicting overall survival

	Univariate analysis		Multivariate analysis	
	HR (95% CI)	*P* value	Adjusted HR^a^ (95% CI)	*P* value
Preoperative NLR				
LNLR (NLR < 2)	Reference		Reference	
HNLR (NLR ≥ 2)	1.719 (1.306-2.262)	0.000	1.449 (1.048-2.004)	0.025
Postoperative NLR				
LNLR (NLR < 1.7)	Reference		Reference	
HNLR (NLR ≥ 1.7)	1.946 (1.410-2.686)	0.000	1.556 (1.112-2.176)	0.010
NLR change				
LL (preoperative NLR < 2, postoperative NLR < 1.7)	Reference		Reference	
LH (preoperative NLR < 2, postoperative NLR ≥ 1.7)	1.403 (0.791-2.490)	0.247	1.040 (0.575-1.882)	0.896
HL (preoperative NLR ≥ 2, postoperative NLR < 1.7)	1.370 (0.894-2.100)	0.148	1.070 (0.692-1.655)	0.761
HH (preoperative NLR ≥ 2, postoperative NLR ≥ 1.7)	2.600 (1.763-3.834)	< 0.001	1.817 (1.220-2.706)	0.003

*HR* hazard ratio, *CI* confidence interval, *NLR* neutrophil-to-lymphocyte ratio, *LNLR* low NLR, *HNLR* high NLR

^a^Adjusted for age, approach method, extent of resection, depth of invasion, node status, stage, and adjuvant chemotherapy

In univariate analyses, age, approach method, extent of resection, depth of invasion, node status, stage, adjuvant chemotherapy, preoperative NLR, and NLR change were significant prognostic factors in terms of DFS. In multivariate analyses, preoperative HNLR, postoperative HNLR, and HH NLR change were not significant risk factors for DFS after adjusting the inflammatory markers for age, approach method, extent of resection, depth of invasion, node status, stage, and adjuvant chemotherapy (Table 3) (*P =* 0.122, 0.513, and 0.143, respectively).

**Table 3:** Univariate and multivariate analyses of factors predicting disease-free survival

	Univariate analysis		Multivariate analysis	
	HR (95% CI)	*P* value	Adjusted HR^a^ (95% CI)	*P* value
Preoperative NLR				
LNLR (NLR < 2)	Reference		Reference	
HNLR (NLR ≥ 2)	1.829 (1.337-2.503)	0.000	1.319 (0.929-1.873)	0.122
Postoperative NLR				
LNLR (NLR < 1.7)	Reference		Reference	
HNLR (NLR ≥ 1.7)	1.224 (0.853-1.757)	0.273	1.136 (0.775-1.664)	0.513
NLR change				
LL (preoperative NLR < 2, postoperative NLR < 1.7)	Reference		Reference	
LH (preoperative NLR < 2, postoperative NLR ≥1.7)	0.795 (0.377-1.676)	0.546	0.764 (0.356-1.642)	0.491
HL (preoperative NLR ≥ 2, postoperative NLR < 1.7)	1.806 (1.201-2.715)	0.005	1.168 (0.765-1.783)	0.472
HH (preoperative NLR ≥ 2, postoperative NLR ≥ 1.7)	1.955 (1.269-3.011)	0.002	1.400 (0.892-2.197)	0.143

*HR* hazard ratio, *CI* confidence interval, *NLR* neutrophil-to-lymphocyte ratio, *LNLR* low NLR, *HNLR* high NLR, *LL* low to low, *LH* low to high, *HL* high to low, *HH* high to high

^a^Adjusted for age, approach method, extent of resection, depth of invasion, node status, stage, and adjuvant chemotherapy

### Overall survival stratified by tumor stage

When the OS of the patients were stratified according to tumor stage, HNLR was significantly associated with poor prognosis in stage I and III but not in stage II disease (Fig. 2b, c, d). The 5-year OS rates of the NLR changes were significantly different in all stages (Table 4).

**Table 4:** Five-year overall survival based on tumor stage and the postoperative NLR

	Stage I		Stage II		Stage III	
	Number	5-YSR (%)	Number	5-YSR (%)	Number	5-YSR (%)
**NLR**						
LNLR	875	97.5	567	88.5	157	67.6
HNLR	352	92.0	237	82.0	50	50.8
***P*** **value**		0.000		0.241		0.006
**NLR change**						
LL (preoperative NLR < 2, postoperative NLR < 1.7)	416	97.1	87	85.1	80	73.8
LH (preoperative NLR < 2, postoperative NLR ≥ 1.7)	93	93.6	14	100.0	16	56.3
HL (preoperative NLR ≥ 2, postoperative NLR < 1.7)	150	98.7	68	92.7	71	60.6
HH (preoperative NLR ≥ 2, postoperative NLR ≥ 1.7)	144	91.0	36	75.0	49	49.0
***P*** **value**		0.002		0.034		0.011

*5-YSR* 5-year survival rate, *NLR* neutrophil-to-lymphocyte ratio, *LNLR* low NLR, *HNLR* high NLR, *LL* low to low, *LH* low to high, *HL* high to low, *HH* high to high

## Discussion

This study examined the associations of postoperative NLR, and its preoperative to postoperative changes, with the prognosis of gastric cancer patients, as well as the clinical utility of these measurements. The results showed that postoperative NLR and changes in NLR are independent prognostic factors for OS in patients with gastric cancer.

Increasing evidence supports an association between cancer and inflammation [19]. In particular, inflammatory processes contribute to cancer initiation, promotion, progression, and invasion [20]. In addition, inflammation is one of the seven main characteristics of cancer [21]. However, the precise mechanism underlying the association between increased NLR and poor long-term outcome in cancer patients is unclear. As NLR depends on two factors (neutrophil and lymphocyte counts), a high NLR may contribute to postoperative prognosis through the following possible mechanism. First, tumor-associated neutrophils may play a role in cancer progression by releasing factors that modulate the extracellular matrix and inflammation in the tumor microenvironment [22]. In particular, they play important roles during the initial angiogenic process in experimental tumor models [23]. Second, lymphocytes are the immune cells most responsible for the body’s protective effector immune response and antitumor response. That is, a decrease in circulating lymphocytes indicates a reduction of immune surveillance, thus enabling tumor growth [24]. The results of our clinical study support previous basic research in the same area.

The main strength of this study was its use of reliable NLR cut-off values, based on a large pool of data for gastric cancer patients. In addition, because a complete blood count is routinely obtained in all cancer patients during follow-up, evaluation of the prognosis of these patients required no additional effort. Thus, our method based on measurements of a systemic inflammatory parameter is a simple, cost-effective, and reproducible technique for assessing the survival of gastric cancer patients. Although the present study had a similar setting to a previous study of preoperative NLR [12], our analysis focused on postoperative NLR and dynamic changes in NLR. It is useful to evaluate the postoperative NLR because it may reflect residual host immune activity [25]. The postoperative systemic inflammatory state plays an important role in preventing tumor recurrence. That is, a postoperative increase in NLR indicates a pro-tumor inflammatory response of the host, whereas a postoperative decrease shows an anti-tumor immune response of the host [6]. For clinicians, therapeutic decision-making during the routine follow-up of high-risk patients after surgery is challenging, where early intervention is crucial to prevent recurrence. In clinical fields, tumor markers such as CEA, CA 19-9, and CA 72-4 are widely evaluated as laboratory follow-up tools. However, there are certain limitations to using these markers to detect recurrence and predict survival after surgery, because these markers have low sensitivity. Marrelli et al. reported sensitivities of 44% for CEA, 56% for CA 19-9, and 51% for CA 72-4 in patients with recurrence [26]. However, the use of postoperative NLR and changes in NLR combined with existing tumor markers may provide a novel postoperative risk stratification tumor marker model.

The prognostic role of preoperative inflammatory markers has been well studied in gastric cancer patients [12–18]. However, the prognostic role of postoperative NLR in such patients has attracted less research attention [5, 9–11]. Some researchers have evaluated the prognosis based on the simple postoperative value [5, 11], while others have evaluated it according to preoperative to postoperative changes in NLR [9, 10]. The prognostic importance of this change reflects a dynamic change in the balance between host inflammatory and immune responses rather than the simple preoperative or postoperative NLRs. In Korea, a few studies have examined the associations between postoperative inflammatory markers, especially the NLR, and the prognosis of patients with gastric cancer. Kim et al. suggested that postoperative NLR predicts long-term recurrence after gastric cancer surgery. However, they used the postoperative day 3 NLR value and did not examine the dynamic change from preoperative to postoperative NLR, unlike our study [5]. Min et al. studied changes in NLR, similar to our study, but they divided the patients into two groups, i.e., negative and positive groups, according to the difference between preoperative and postoperative values. In addition, the postoperative NLR evaluation was performed at 3 to 6 months after surgery [10]. To the best of our knowledge, this is the first study to demonstrate the prognostic value of both the postoperative NLR and NLR change in Korean gastric cancer patients. Another strength of our study was that these two prognostic indicators were identified using the same data.

Despite its strengths, our study also had some limitations. First, we performed retrospective analysis of data collected from a single institution. Second, some bias can occur, because our study enrolled the patients even if there was recurrence within 6 months after operation. Thus, authors tried to perform additional analyses excluding recurrence within 6 months after operation. The results were shown in Additional file 1: Supplement table. The trend was similar with our original study described in the “Result” section except for non-significant *P* value of the preoperative NLR in multivariate analysis for OS. Third, we did not evaluate changes in other inflammatory markers such as CRP, procalcitonin, PLR, and neutrophil-to-platelet ratio (NPR). Further studies are needed to validate the significance of our postoperative risk stratification model including NLR and other inflammatory markers, including CRP, procalcitonin, PLR, and NPR. Fourth, our study only demonstrated the postoperative 6-month NLR after surgery due to its retrospective nature. There is no consensus regarding the appropriate timing for measurement of postoperative inflammatory markers as prognostic predictors, with time points to check postoperative NLR ranging from 3 days to 6 months in previous gastric cancer studies [5, 9, 10]. Surgery is a major event that induces an acute inflammatory response [27]. The acute inflammatory reaction caused by surgery resolves within a short time, and its effects on the tumor disappear completely by several months postoperatively. Although there have been no reports regarding the exact time when inflammation due to the surgical wound healing process ceases, several groups have suggested that 1 month postoperatively is an appropriate check-up point [28, 29]. Further studies are required to determine the optimal check-up time point for reflecting postoperative NLR as a prognostic predictor in gastric cancer patients.

## Conclusion

Preoperative NLR, postoperative NLR, and changes in NLR were significant prognostic factors for OS in patients with gastric cancer. Patients with high preoperative and postoperative NLR, and especially with persistent elevation of preoperative to postoperative NLR, had poor prognosis with regard to OS. Based on these results, we suggest that the NLR should be included in the routine postoperative assessment of patients with gastric cancer. The identification of high-risk patients will allow early interventions that may reduce the risk of recurrence and improve oncological outcomes.

## Supplementary Information

**Additional file 1: Table S1.** Associations of patient characteristics with the postoperative neutrophil-to-lymphocyte ratio (NLR).

## Data Availability

Access to the database may be obtained from the corresponding author on reasonable request.
