# Supplementary material for: The preoperative and the postoperative neutrophil-to-lymphocyte ratios both predict prognosis in gastric cancer patients
Source: World J Surg Oncol. 2020 Nov 10;18:293. doi: 10.1186/s12957-020-02059-4 (PMC7656697; doi:10.1186/s12957-020-02059-4)
Supplement: Supplementary file 1 — Additional file 1: Table S1. Associations of patient characteristics with the postoperative neutrophil-to-lymphocyte ratio (NLR). [file 12957_2020_2059_MOESM1_ESM.doc]

Table 1. Associations of patient characteristics with the postoperative neutrophil-to-lymphocyte ratio (NLR)

| Factors | NLR | | *P* value | |  |
| --- | --- | --- | --- | --- | --- |
|  | LNLR  n=875(%) | HNLR  n=352(%) |  |  | |
| Age in years, (mean±SD) | 57.4±11.7 | 60.7±11.7 | <0.001 | |  |
| Gender |  |  | 0.015 | |  |
| Male | 556 (64.0) | 242 (71.4) |  | |  |
| Female | 313 (36.0) | 97 (28.6) |  |  |  |
| Approach method |  |  | 0.630 | |  |
| Open | 541 (62.3) | 207 (61.1) |  | |  |
| Laparoscopy | 309 (35.6) | 127 (37.5) |  |  |  |
| Robot  Extent of resection | 19 (2.2) | 5 (1.5) | 0.103 | |  |
| Partial gastrectomy | 640 (73.6) | 265 (78.2) |  | |  |
| Total gastrectomy | 229 (26.4) | 74 (21.8) |  |  |  |
| Histologic type |  |  | 0.001 | |  |
| Differentiated | 384 (44.2) | 186 (54.9) |  | |  |
| Undifferentiated | 485 (55.8) | 153 (45.1) |  |  |  |
| Depth of invasion* |  |  | 0.291 | |  |
| T1 | 542 (62.4) | 218 (64.3) |  | |  |
| T2 | 87 (10.0) | 43 (12.7) |  |  |  |
| T3 | 119 (13.7) | 39 (11.5) |  |  |  |
| T4 | 121 (13.9) | 39 (11.5) |  |  |  |
| Node status* |  |  | 0.269 | |  |
| N0 | 586 (67.4) | 238 (70.2) |  | |  |
| N1 | 118 (13.6) | 34 (10.0) |  |  |  |
| N2 | 89 (10.2) | 31 (9.1) |  |  |  |
| N3 | 76 (8.7) | 36 (10.6) |  |  |  |
| Stage* |  |  | 0.187 | |  |
| I | 565 (65.0) | 236 (69.6) |  | |  |
| II | 157 (18.1) | 47 (13.9) |  |  |  |
| III | 147 (16.9) | 56 (16.5) |  |  |  |
| Adjuvant chemotherapy |  |  | 0.019 | |  |
| No | 597 (68.7) | 256 (75.5) |  | |  |
| Yes | 272 (31.3) | 83 (24.5) |  | |  |

*According to the AJCC TNM classification, 7th edition.

SD, standard deviation.

Table 2. Univariate and multivariate analyses of factors predicting overall survival

|  | Univariate analysis | | Multivariate analysis | | |
| --- | --- | --- | --- | --- | --- |
|  | HR(95% CI) | *P* value | | Adjusted HR†  (95% CI) | *P* value |
| Preoperative NLR |  |  | |  |  |
| LNLR (NLR<2) | Reference |  | | Reference |  |
| HNLR (NLR≥2) | 1.606 (1.151-2.240) | 0.005 | | 1.374 (0.981-1.924) | 0.064 |
| Postoperative NLR |  |  | |  |  |
| LNLR (NLR<1.7) | Reference |  | | Reference |  |
| HNLR (NLR≥1.7) | 1.777 (1.265-2.497) | 0.001 | | 1.522 (1.072-2.160) | 0.019 |
| NLR change |  |  | |  |  |
| LL  (preoperative NLR<2, postoperative NLR<1.7) | Reference |  | | Reference |  |
| LH  (preoperative NLR<2, postoperative NLR ≥1.7) | 1.367 (0.758-2.467) | 0.299 | | 1.100 (0.595-2.032) | 0.761 |
| HL  (preoperative NLR≥2, postoperative NLR <1.7) | 1.293 (0.833-2.009) | 0.253 | | 1.054 (0.671-1.656) | 0.819 |
| HH  (preoperative NLR ≥2, postoperative NLR ≥1.7) | 2.263 (1.503-3.408) | <0.001 | | 1.732 (1.141-2.629) | 0.010 |

HR, hazard ratio; CI, confidence interval; NLR, neutrophil-to-lymphocyte ratio; LNLR, low NLR; HNLR, high NLR.

*According to the AJCC TNM classification, 7th edition.

†Adjusted for age, approach method, extent of resection, depth of invasion, node status, stage, and adjuvant chemotherapy

Table 3. Univariate and multivariate analyses of factors predicting disease-free survival

|  | Univariate analysis | | | Multivariate analysis | |
| --- | --- | --- | --- | --- | --- |
|  | HR(95% CI) | *P* value | | Adjusted HR  (95% CI) | *P* value |
| Preoperative NLR |  | |  |  |  |
| LNLR (NLR<2) | Reference | |  | Reference |  |
| HNLR (NLR≥2) | 1.644 (1.136-2.379) | | 0.008 | 1.147 (0.789-1.667) | 0.472 |
| Postoperative NLR |  | |  |  |  |
| LNLR (NLR<1.7) | Reference | |  | Reference |  |
| HNLR (NLR≥1.7) | 0.880 (0.574-1.350) | | 0.559 | 0.920 (0.597-1.418) | 0.709 |
| NLR change |  | |  |  |  |
| LL  (preoperative NLR<2, postoperative NLR<1.7) | Reference | |  | Reference |  |
| LH  (preoperative NLR<2, postoperative NLR ≥1.7) | 0.519 (0.207-1.305) | | 0.163 | 0.575 (0.226-1.462) | 0.245 |
| HL  (preoperative NLR≥2, postoperative NLR <1.7) | 1.627 (1.059-2.498) | | 0.026 | 1.060 (0.686-1.638) | 0.794 |
| HH  (preoperative NLR ≥2, postoperative NLR ≥1.7) | 1.358 (0.826-2.233) | | 0.227 | 1.090 (0.659-1.802) | 0.738 |

HR, hazard ratio; CI, confidence interval; NLR, neutrophil-to-lymphocyte ratio; low NLR; HNLR, high NLR; LL, low to low; LH, low to high; HL, high to low; HH, high to high.

* According to the AJCC TNM classification, 7th edition.

† Adjusted for approach method, extent of resection, depth of invasion, node status, stage, and adjuvant chemotherapy
